# Understanding the spatio-temporal behaviour of the sunflower crop for subfield areas delineation using Sentinel‐2 NDVI time-series images in an organic farming system

**DOI:** 10.1016/j.heliyon.2023.e19507

**Published:** 2023-08-30

**Authors:** Stefano Marino

**Affiliations:** Department of Agricultural, Environmental and Food Sciences (DAEFS), University of Molise, Via De Sanctis, I-86100, Campobasso, Italy

**Keywords:** Yield, Cluster analysis, K-means, Remote sensing, Semi-automatic classification

## Abstract

The study investigates the suitability of time series Sentinel-2 NDVI-derived maps for the subfield detection of a sunflower crop cultivated in an organic farming system. The aim was to understand the spatio-temporal behaviour of subfield areas identified by the K-means algorithm from NDVI maps obtained from satellite images and the ground yield data variability to increase the efficiency of delimiting management zones in an organic farming system.

Experiments were conducted on a surface of 29 ha. NDVI time series derived from Sentinel-2 images and k-means algorithm for rapidly delineating the sunflower subfield areas were used. The crop achene yields in the whole field ranged from 1.3 to 3.77 t ha^−1^ with a significant within-field spatial variability. The cluster analysis of hand-sampled data showed three subfields with achene yield mean values of 3.54 t ha^−1^ (cluster 1), 2.98 t ha^−1^ (cluster 2), and 2.07 t ha^−1^ (Cluster 3). In the cluster analysis of NDVI data, the k-means algorithm has early delineated the subfield crop spatial and temporal yield variability. The best period for identifying subfield areas starts from the inflorescences development stage to the development of the fruit stage. Analyzing the NDVI subfield areas and yield data, it was found that cluster 1 covers an area of 42.4% of the total surface and 50% of the total achene yield; cluster 2 covers 35% of both surface and yield. Instead, the surface of cluster 3 covers 22.2% of the total surface with 15% of achene yield. K-means algorithm derived from Sentinel-2 NDVI images delineates the sunflower subfield areas.

Sentinel-2 images and k-means algorithms can improve an efficient assessment of subfield areas in sunflower crops. Identifying subfield areas can lead to site-specific long-term agronomic actions for improving the sustainable intensification of agriculture in the organic farming system.

## Highlights

• Organic farming for reducing the environmental impact
• The growing demand for stable crop production requires useful tools for the sustainable intensification of agriculture
• Combining high-resolution Sentinel-2 imagery and cluster analysis for subfield areas delineation
• Identification of subfiles areas starts from the Inflorescences development stage
• Subfield area delineation for site-specific long-term agronomic actions

## Graphical abstract

**Figure:**
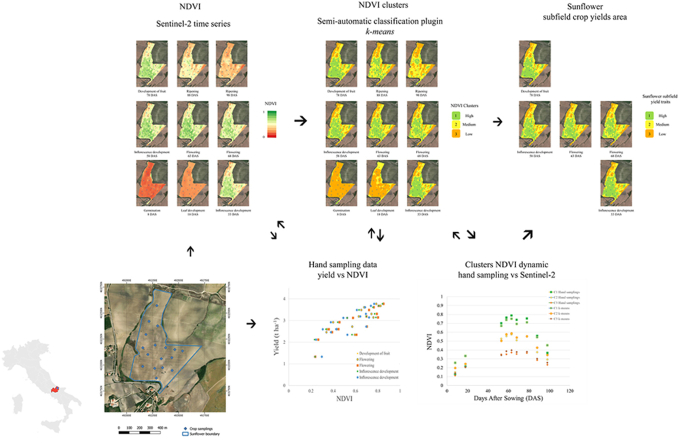

## 1 Introduction

Producing wholesome food for the world's expanding population is agriculture's main goal. Due to its impact on biodiversity, climate change, and public health, modern farming has come under scrutiny [1,2]. Agriculture must meet the challenges of feeding a growing population while minimizing its global environmental impacts [[3], [4], [5]]. A radical alternative to conventional agriculture is organic farming, which emphasizes diversifying and rotating crops, managing pests naturally, and improving the soil health and avoiding synthetic chemical fertilizers and pesticides; it combines traditional conservation farming methods with modern farming technologies [6]. Organic farming provides healthy food [7], conserves species richness, and maintains ecosystem functioning [8]. Organic agriculture products are healthier than conventional products and in increasing demand; furthermore organic farming is more respectful for the environment and sustainability [9]. Due to different management techniques, organic fields differ from conventional ones in a number of ways, the most notable of which are lower yields, slower crop growth rates and biomass, lower plant nitrogen and chlorophyll concentrations, higher soil organic matter content and crop spatial variability [6,10,11]. The adaptability and stability of yield are one of the most important requirements in sustainable agriculture [12]. There is now a revitalized interest in boosting yields in organic farming system to provide more organic food for a growing, and reduce negative impacts per unit produced [13]. Furthermore, increasing food production by reducing yield gaps in a changing climate and reducing markedly the environmental footprint of agriculture is a challenge [5]. Sunflower (*Helianthus annuus* L.) is an important oil crop of the Mediterranean region [14]. Cultivars rich in oleic acid prevent cardiovascular diseases and thus represent an essential dietary source for humans. After soybean, Sunflower ranks second in the world for oil consumption due to its high oil content (36%–55%) [15]. The sunflower is a drought-tolerant species, cultivated in the Mediterranean regions as spring-summer crop. Sunflower is a valid option for arid areas where water resources (used for irrigation) are decreasing and the risk of water deficit is expected to increase [16]. The sunflower requires between 350 and 1500 mm of rain per year, with monthly temperatures ranges from 15 to 39 °C between April and September. Debaeke et al. [17], in a study on the Sunflower crop and climate change: state that according to all climatic models, sunflowers will continue to be potentially grown in over 60% of southern Europe (35–44° N). Due to their ability to grow in different agroecological conditions and moderate drought tolerance, sunflowers may become an oil crop of preference, particularly considering global environmental changes [18]. Different studies have highlighted the high reliance of this crop on sufficient water availability during all its growth phases [19,20]. However, many studies on the sunflower showed that the Sunflower biomass was 40% lower and the achene yield was 30% lower in the organic than in the conventional system [21]. Precision Agriculture aims to maximize the efficiency of crop system management by defining areas to be managed homogeneously within a field [22,23]. Indeed, the availability of remote sensing data of Sentinel-2 satellite with multiple spectral bands in the near-infrared (NIR) and red is making remote sensing a useful tool for crop growth monitoring and stress detection, crop phenology and variability as well as environmental monitoring and crop yield prediction [[24], [25], [26]]. Among the vegetation indices, seasonal NDVI images are the most commonly used data for building regression models of crop yield [27]. Based on vegetation indices and crop yield data, a number of strategies have been developed to forecast the sunflower crop yields at field and regional scales [15,[28], [29], [30], [31], [32]]. However, increasing yields in organic agriculture need to identify yield gaps and subfield areas for how to yield gaps may be closed at the farm level [33] (Lobell, 2013). The difference between the potential yield and the actual yield is defined as yield gap [[23], [24], [25], [26], [27], [28], [29], [30], [31], [32], [33], [34]]. Mapping the within-field crop variability is crucial in precision agriculture, which seeks to balance agronomic strategies with spatial crop demands to manage crops better [22,35,36]. Satellite imagery and delineating management zones based on remote sensing play a key role [35]. Remote sensing from satellites can provide high-resolution and reasonably priced spatio-temporal data that can precisely identify management zones [37,38]. Unsupervised classification algorithms are often used to divide a field into zones [39]. Cluster analysis is an important method in precision agriculture to define management zones [38,40,41]. K-means is one of the most used methods for the definition of management zones (MZs) in agricultural fields, while K-means methods may not be adequate for some fields [40], satisfactory results using K-means have been reported [36,[42], [43], [44]]. Currently, most studies focus only on forecast yield and site-specific agrochemical distribution, and few studies combine remote sensing data analysis with land yield components in the organic farming system [45]. Introduced methods of digital agriculture (e.g., technologies that enable precision agriculture) may provide a solution that balances enhanced yield levels and environmental friendliness [46]. However, the potential of precision agriculture technologies in organic farming systems has yet to be widely studied [47].

The study investigates the suitability of time series Sentinel-2 NDVI-derived maps for the subfield detection of a sunflower crop cultivated in an organic farming system. QGIS Semi‐automatic classification and k-means algorithm for rapidly delineating the sunflower subfield areas using Sentinel‐2 satellite imagery was used. Sentinel-2 NDVI-derived maps were related to ground sampling yield and yield component data. The aim was to understand the spatio-temporal behaviour of subfield areas identified by the K-means algorithm from NDVI maps obtained from satellite images and the ground yield data variability to increase the efficiency of delimiting management zones in an organic farming system.

## 2 Materials and methods

### 2.1 Study area

**Fig. 1:**
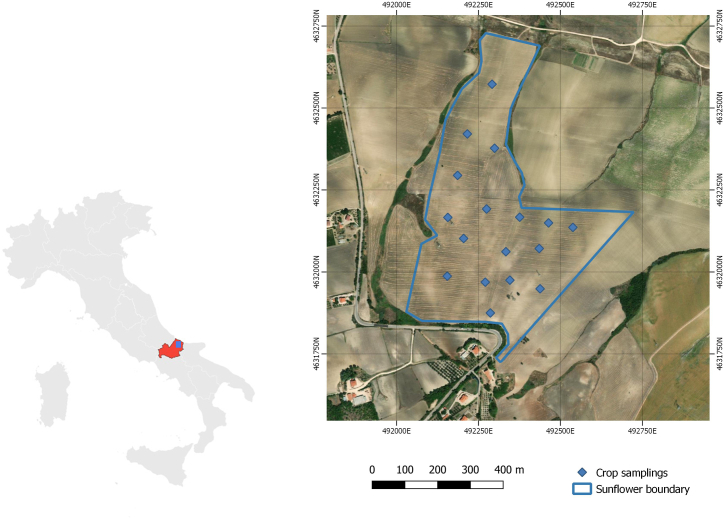
Study area, sunflower boundary and crop samplings (RGB image).

The experiment was carried out in Central Italy (492680.30 E, 4632601.7 N; UTM-WGS84 zone 33 N Italy) on a surface of 29 ha. The experimental site was located in Larino, Molise region, (Fig. 1). A Mediterranean climate characterizes the growing area. A meteorological station recorded the meteorological data on the farm from May 2020 to August 2021.The total rainfall were 158 mm and mean temperature 20.5 °C. The minimum temperature was recorded in May (7 °C) and the maximum in July (40 °C). The mean air temperature in 2021 was 14 °C, and the annual precipitation was 700 mm, concentrated in autumn and winter.

### 2.2 Ground data measurements - crop traits and phenological stages

The soil is a calcareous clay soil; a harrow breaker ploughs it, and after ploughing, a disc plough, a grubber (2) and a Vibro cultivator, were used for preparing the seed bed. A total amount of 75000 seeds per hectare of Sunflower (*Helianthus annuus* L.*)* P64HE133 (Pioneer) medium early sunflower seed maturity, *sclerotinia* resistant and *phoma* resistant, was used, sowing date was 5 May and harvest data 29 August. The preceding crop was the broad beans (*Vicia Faba minor* L.).

On 29 August, the field was harvested mechanically (117 days after sowing), and the achene yield was determined at 13% moisture content. On August 24th^,^ 2021, 16 georeferenced samples from 1 m^2^ were gathered to weigh and measure yield and yield-related traits. Crop yield, biomass, stem diameter, plant height, achene yield, and head diameter were collected. Yield and dry biomass were determined after oven-drying plant material at 65 °C for 72 h. The crop density was recorded by counting the number of plants in a sampling surface of 1 m^2^ per georeferenced sample. The phenological stages of the sunflower were periodically recorded according to the BBCH-scale [48]**.** In this scale, the sunflower plant has eight main stages. Sowing typically takes place from the third week of April to the first week of May. the harvest usually takes place about 4 months after sowing.

### 2.3 Sentinel-2 data

Sentinel-2 satellites (2A and 2B), (Copernicus Programme) launched by the ESA (European Space Agency) provide multispectral data (13 bands) with spatial resolution of 10 m, 20 m and 60 m [49] (Mecklenburg et al., 2012). The revisit period was 5. This study used radiometrically and atmospherically corrected Level 2A, BOA (bottom-of-atmosphere) (BOA) and close to zero (<10%) cloud cover reflectance products. Nine cloud-free Sentinel-2A images from 12 May to 10 August 2021 (Table 1) were downloaded from ESA's Copernicus Open Access Hub [50].

**Table 1:** Sentinel-2 available cloud free image data, Acquisition period, Sunflower phenology (crop growth stage, BBCH scale (Meier 2001) and Day After Showing (DAS).

Sentinel-2 images	Acquisition period	Crop Growth Stage BBCH scale (Meier 2001)	DAS
L2A_T33TVG_A021698_20210502T095025	May 12, 2021	Germination	8
L2A_T33TVG_A021984_20210522T095029	May 22, 2021	Leaf Development	18
L2A_T33TVG_A031393_20210626T095357	June 26, 2021	Inflorescence development	53
L2A_T33TVG_A022556_20210701T095030	July 01, 2021	Inflorescence development	58
L2A_T33TVG_A031536_20210706T095401	July 06, 2021	Flowering	63
L2A_T33TVG_A022699_20210711T095031	July 11, 2021	Flowering	68
L2A_T33TVG_A022842_20210721T095031	July 21, 2021	Development of fruit	78
L2A_T33TVG_A022985_20210731T095031	July 31, 2021	Ripening	88
L2A_T33TVG_A023128_20210810T095030	August 10, 2021	Ripening	98

SNAP-ESA Sentinels Application Platform v.6.0.4 [51] was used to resample the images to a resolution of 10 m.

The NDVI time series were computed from red (ρRED) and near-infrared (ρNIR) spectral bands, as follows [52]:

NDVI = (ρNIR – ρRED)/(ρNIR + ρRED)

The procedure for the NDVI computation and subfield areas detection was reported in section 2.4 Statistical analysis.

### 2.4 Statistical analysis

Sentinel-2 spectral bands n. 4 (Red) with a Central wavelength (nm) of 665 and bandwidth 30 and n. 8 (NIR) Central wavelength (nm) of 842 and bandwidth 115 were elaborated in QGIS software (Quantum GIS), provided by the Open Source Geospatial Foundation (OSGeo).

The QGIS 'Raster Calculator' tool, was used for the NDVI computation. Furthermore, the Point Sampling Tool was used to extract raster NDVI data values for each georeferenced point. The Semi-automatic classification plugin and the k-means cluster method were used for categorizing the NDVI images [53]. The Semi-Automatic Classification Plugin with built-in algorithms developed in Python and third-party algorithms for Sentinel-2 pre-processing of images and the post-processing of classifications through ESA SNAP was used [51]. The algorithm splits clusters with too high a variability of spectral signatures based on the standard deviation since many spectral signatures can be defined in the initial pre-processing phase to capture the number of clusters expected in the image as reported by Congedo [54].

The crop yield and yield traits were analyzed using hierarchical clustering Ward's minimum variance approach [55], as reported in a previous paper by Marino et al. [56]. Finding a system that can categorize data in which the group members share properties is the goal of cluster analysis. The first step in aggregative clustering is to combine the two groups that are the most similar based on the distance matrix. Up until every sample has been incorporated to a single big cluster, this process is repeated. A distance criterion is used to determine the final split [57]. The researcher decides to terminate the agglomeration process when succeeding clusters are too far apart to merge, starting at the bottom of the dendrogram. In this study, we selected a significant number of clusters using the scree plot [58].. The yield-related features were used to build the clusters, and statistical tests were employed to confirm (a posteriori) dependency conditioning of the various variables. The Kruskal–Wallis test [59] is most commonly used when there is one nominal variable and one measurement variable which does not meet the normality assumption. The Kruskal–Wallis test verifies whether three or more independent groups have the same distribution. Statistical procedures were computed using STATISTICA (StatSoft., Inc., Tulsa, OK, USA). Statistical procedures were computed using OriginPRO 8 (Origin Lab Corporation, Northampton, MA 01060, USA).

Regression analysis, coefficients of determination, significance levels, and RMSE were computed on two sets of geo-referred data: ground crop samplings and NDVI data using the statistical package Origin PRO 8 (Origin Lab Corporation, Northampton, MA, USA).

## 3 Results

### 3.1 Sentinel-2 NDVI-derived time-series spatial and temporal variability

**Fig. 2:**
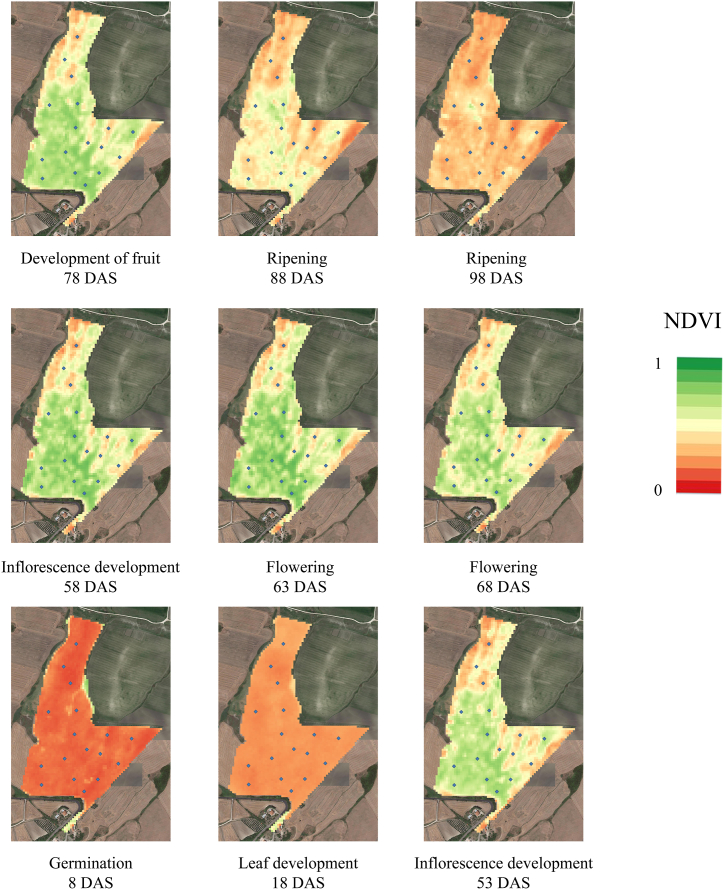
NDVI sentinel-2 images (range 0–1). Images were collected at Germination (8 DAS), Leaf Development (18 DAS), Inflorescence development (53 and 58 DAS), Flowering (63 and 68 DAS), Development of fruit (78 DAS) and Ripening (88 and 98 DAS) stages.

NDVI images Sentinel-2 (range 0–1) were collected at 9 different Days After Sowing (DAS), starting from germination (8 DAS) to ripening stage 98 DAT according to the BBCH scale and reported in Fig. 2. The images showed the NDVI spatial and temporal variability dynamics. The NDVI value at germination and leaf development showed the lowest range between minimum and maximum values of 0.19 and 0.23, respectively; in contrast, from inflorescence development (53 DAS) to the ripening stage (78 DAS), the NDVI range was from 0.57 to 0.71, with the highest range at the flowering stage and the lowest at the ripening stage (Table 2). The highest NDVI values were recorded from the inflorescence stage to the development of fruit (from 53 to 78 DAS). Moreover, the mean value of NDVI in the field was higher than 0.5 from the inflorescence stage to the development of fruit. From germination to leaf development and at the ripening stage, the NDVI mean value was lower, ranging from 0.15 at the germination stage (8 DAS) to 0.43 at the ripening stage (88 DAS). From a visual assessment (Fig. 2), between the inflorescence development and the development of fruit, a central area of the field with higher NDVI values is easily identifiable. Furthermore, an area in the upper part and on the right side of the field with lower NDVI values was detected. No differences were highlighted in the early stages (8 and 18 DAS), and at the ripening stage, especially at 98 DAS (20 days before the harvest).

**Table 2:** Minimum, maximum, mean value, range, standard deviation (SD) and Sum of the square of NDVI data derived from Sentinel-2 images at different Sunflower crop growth stages (BBCH scale) and Days After Sowing (DAS).

		NDVI					
Crop Growth Stage BBCH scale (Meier, 2001)	DAS	Min	Max	Range	Mean Value	SD	Sum of the squares
Germination	8	0.09	0.28	0.19	0.15	0.07	19.0
Leaf Development	18	0.17	0.4	0.23	0.22	0.02	1.65
Inflorescence development	53	0.16	0.79	0.63	0.54	0.14	56.9
Inflorescence development	58	0.18	0.86	0.68	0.60	0.15	63.2
Flowering	63	0.16	0.87	0.71	0.61	0.15	61.5
Flowering	68	0.19	0.84	0.65	0.56	0.13	48.8
Development of fruit	78	0.18	0.81	0.63	0.59	0.14	54.4
Ripening	88	0.15	0.75	0.60	0.43	0.10	29.8
Ripening	98	0.12	0.69	0.57	0.32	0.07	14.7

### 3.2 Sub-field sunflower by NDVI k-means clustering

**Fig. 3:**
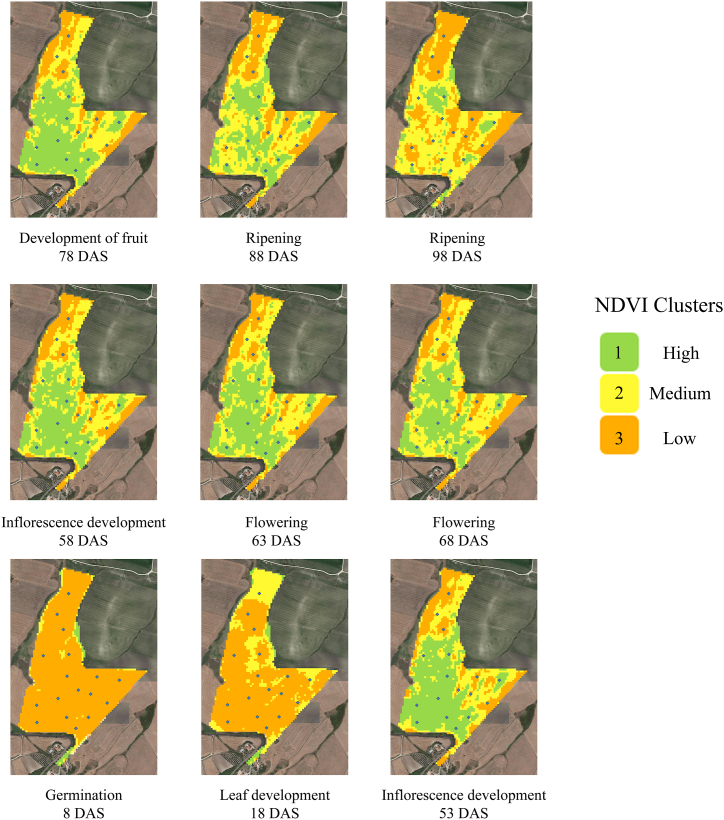
NDVI sentinel-2 Clustered images (High, Medium and Low). Data were clustered by the semi-automatic classification plugin and k-means algorithm. Images were collected at Germination (8 DAS), Leaf Development (18 DAS), Inflorescence development (53 and 58 DAS), Flowering (63 and 68 DAS), Development of fruit (78 DAS) and Ripening (88 and 98 DAS) stages.

**Fig. 4:**
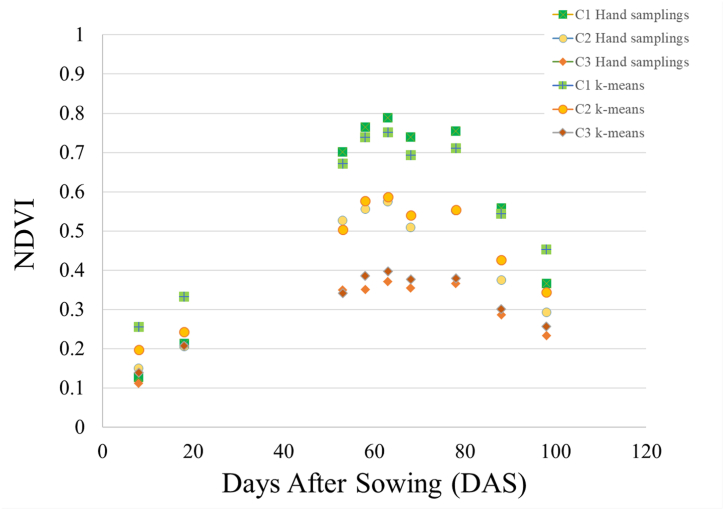
Hand sampling NDVI-related cluster data and Sentinel-2 NDVI k-means cluster at different crop growth stages.

The NDVI images collected at different crop stages were clustered by QGIS k-means semi-automatic classification plug-in. Fig. 3 reports the spatial and temporal variability evolution of the identified clustered areas. No significant differences were recorded at germination (8 DAS), and no subfield areas were identified; therefore, one NDVI cluster was identified. At Leaf Development (18 DAS), two clusters were identified; however, the clusters did not show any agronomically relevant information. Three significant subfield areas were identified from inflorescence development (53 DAS) to the ripening stage (98 ZS). The three discovered clusters were categorized according to Cluster 1 (High NDVI values), Cluster 2 (Medium NDVI values) and Cluster 3 (Low NDVI values) (Fig. 4). The NDVI mean values from inflorescence development (53 DAS) to development of fruit (78 DAS) range from 0.34 to 0.38 for the cluster 3 area, from 0.50 to 0.58 for the cluster 2 area and from 67 to 0.75 for the cluster 1 (high NDVI values). At the ripening stage (88–98 DAS), the NDVI mean values of 0.28 Cluster 3 (Low), 0.39 Cluster 2 (Medium) and 0.50 Cluster 1 (High) were recorded.

Two areas with low values of NDVI (Orange) were detected starting from the inflorescence development stage (53 DAS). The NDVI-derived sentinel-2 map clustered showed a surface of cluster 3 area from inflorescence development to development of fruit of about 6.5 ha; it is located on the upper part of the field and the right side of the field. The cluster 2 area showed a mean surface from the inflorescence development stage of 10.3 ha (time series variation ranging from 9.2 to 11.7). All the border areas between the most productive (cluster 1) and the least productive (cluster 3) areas have important areas related to cluster 2 (yellow). Furthermore, other small areas are present throughout the plot. Cluster 1 covers an area of 12.4 Ha, with a dynamic time series variation ranging from 10.9 to 13.5 Ha. The most productive area is located in the central part of the field.

### 3.3 Yield and yield component: whole field and subfield areas analysis

The Sunflower field of 29 ha was harvested on 29 August; the average achene yield was 2.75 t ha^−1^. Crop achene yields in the entire field ranged from 1.3 to 3.77 t ha^−1^, with significant within-field yield variation and spatial heterogeneity observed.^1^. The dry biomass mean value was 852 g m^−2^, biomass ranging from 340 g m^−2^ to 1233 g^−2^, the mean head diameter 13 cm ranging from 7 to 21, the number of plants per square meter ranging from 2 to 8, the stem diameter 2.15 cm, and the average plant height was 120 cm. Georeferenced yield and yield components traits data collected at harvest were clustered to detect group data with significant differences. Three clusters were identified by scree plot analysis. The cluster differences were significant for all crop traits except the number of plants per square meter and height (Table 3). Achene yield and yield traits increased, from the lowest values of the 3rd cluster to the highest values of the 1st cluster. The achene yield of cluster 1 was 354 g m^−2^ (3.54 t ha^−1^) with a mean biomass of about 1120 g m^−2^, and a head diameter 16.4 cm. With an average achene yield of 298 g m^−2^ (2.98 t ha^−1^), lower biomass, and smaller head and stem diameters than cluster 1, cluster 2 displayed lower yield and yield trait values than cluster 1. Cluster 3 showed a reduction of yield and yield traits related to cluster 1 and cluster 2 with mean achene yields of 207 g m^−2^ (2.07 t ha^−1^) and a reduction of the yield components values in the same proportion.

**Table 3:** Crop traits mean values and standard deviations of clusters identified by Ward's cluster method at ripening stage. Analysis of variance between SS, Within SS, F value, *p-value* and Kruskal-Wallis one-way nonparametric test was reported.

			Cluster			
			2		3	
Crop traits rowhead	Mean	±S.D.	Mean	±S.D.	Mean	±S.D.
Achene Yield (g m-2) rowhead	354	(24.4)	298	(25.5)	207	(5.96)
Biomass (g m-2) rowhead	1119	(92.2)	837	(70.8)	571	(161)
Plant number m-2 rowhead	3.75	(1.5)	4.40	(1.08)	5.38	(1.77)
Head diameter (cm) rowhead	16.4	(3.78)	13.3	(3.25)	9.62	(1.75)
Stem diameter (cm) rowhead	2.58	(0.37)	2.22	(0.41)	1.53	(0.36)
Height (cm) rowhead	126.7	(11.1)	121.9	(11.3)	106.6	(19.3)

Analysis of variance				Cluster				
	Between SS	df		Within SS	df	F	p-value	*Kruskal-Wallis one-way*
Achene Yield (g m-2)	48655	2		14057	13	22.5	6.00E-05	**
Biomass (g m-2)	674834	2		142221	13	30.8	1.16E-05	**
Plant number m-2	7.3	2		30.2	13	1.58	0.243	n.s.
Head diameter (cm)	103	2		129	13	5.19	0.0221	*
Stem diameter (cm)	2.48	2		1.96	13	8.23	0.0049	**
Height (cm)	968	2		2381	13	2.64	0.109	n.s.
								

### 3.4 NDVI time-series and yield traits data

The time-series NDVI maps data were related to georeferenced hand-sampled crop yield data. A regression analysis between crop yield and biomass attributes measured at harvest and NDVI data at various growth stages was conducted. (Table 4). No significant regression was found at germination (8 days after sowing (DAS) and leaf development stages (18 DAS) for all crop traits. A significant and favorable regression was revealed by the regression analysis between NDVI vs achene yield and biomass in the 7 sampling dates from inflorescence development (53 DAT) to ripening (47 ZS) with an R^2^ value ranging from 0.58 to 0.73. The highest positive regression was found for yield from 58 DAT (inflorescence development) to 68 DAT (flowering stage), with R^2^ values ranging from 0.6 to 0.57 and for biomass starting from 58 DAT (inflorescence development) to Ripening stage (88 DAT).

**Table 4:** Linear regression value (R^2^) and Root Mean Square Error (RMSE) of NDVI derived from Sentinel-2 and Achene yield and Biomass at differtent Sunflower crop growth stages (BBCH scale) and Day After Sowing (DAS).

		Achene yield		Biomass	
Crop Growth Stage BBCH scale (Meier, 2001)	DAS	R^2^	RMSE	R^2^	RMSE
Germination	8	0.02	66.7	0.01	242.6
Leaf Development	18	0.01	66.9	0.03	240.4
Inflorescence development	53	0.63	40.9	0.61	151.9
Inflorescence development	58	0.71	36.1	0.68	137.6
Flowering	63	0.73	34.5	0.72	128.3
Flowering	68	0.72	35.6	0.69	135.1
Development of fruit	78	0.69	37.1	0.68	137.5
Ripening	88	0.63	41.0	0.69	135.4
Ripening	98	0.58	43.4	0.65	143.1

### 3.5 Sub-field sunflower yield areas

The achene yields georeferenced data were used to analyze the sunflower yield for each cluster area according to NDVI time-series k-means images. The yield for cluster 3 was 13.4 t for 6.5 Ha, 30.7 t for 10.3 Ha for cluster 2 and 43.8 t for 12.4 Ha for cluster 1. The total average yield from inflorescence development to the development of fruit was 3.01 t ha^−1^, ranging from 2.98 to 3.05 t ha^−1^, with an overestimation of about 10% related to the on-field harvest achene yield data. Fig. 4 reports the NDVI mean values of each cluster elaborated starting from Sentinel-2 time-series, and ground truth georeferenced sampling NDVI-related data. There is an important correspondence between the mean NDVI cluster area values and the average values found by the NDVI-related ground truth samplings data starting from the inflorescence development stage. The NDVI values extrapolated by the ground samples clustered yield data showed a difference lower than 10% related to the mean value of the NDVI cluster areas identified by the k-means algorithm from inflorescence development to the development of fruit.

## 4 Discussion

### 4.1 Subfield areas delineation and within-field variability

Understanding the fundamental mechanisms that cause crop stress and yield loss can improve agricultural production's resistance to weather variability and climate change [60]. Röös et al. [13], in a review of the risk and opportunity of increasing yield in organic farming, highlighted that, to be a driving force for increased food system sustainability, organic agriculture may need to reconsider certain fundamental principles. Precision farming or precision agriculture can be one tool for increasing yields in organic agriculture by understanding the subfield crop yields area, the spatio-temporal dynamic and subfield crop stability [22]. Amankulova et al. [15], Fieuzal et al. [31], Dicu et al. [61], found that time-series analysis of Sentinel-2 satellite images can successfully predict sunflower crop yield at the landscape level. The proposed study evaluates the potential of using multi-temporal Sentinel-2A time series optical images to identify subfield areas in an organic farming system. On the basis of a vegetation index derived from satellite data, subfield areas on the field were identified using an unsupervised classification technique (k-means). NDVI maps derived from sentinel-2 images (range 0–1) were collected at 9 different DAS starting from germination (8 DAS) to Ripening stage 98 DAT according to the BBCH scale. The NDVI value at germination and leaf development showed the lowest NDVI values and the lowest NDVI range between minimum and maximum values; it was about 70% lower than that found at the flowering stage (63 DAS). The flowering stage also showed the highest mean and maximum NDVI values. However, the maximum, minimum, and range of NDVI values between the inflorescence development and the development of fruit were very close to those of flowering (about −10%). After the development of fruit, was registered a descending distribution of values, according to Dicu et al. [61](2021). Furthermore, the Sentinel-2 NDVI-derived data distribution during the crop cycle on the sunflower showed the same distribution reported by Narin et al., [62]. The visual assessment of NDVI maps showed, between the inflorescence development and the development of fruit, a central area of the field with higher NDVI values and an area in the upper part and on the right with lower NDVI values. However, these differences cannot lead to a clear identification of significantly different agronomic subfield areas. In the early stages (8 and 18 DAS) of development, there is a lack of visual evidence that could determine the spatial variability of the NDVI. At the ripening stage, the NDVI values are lowered, making interpretation more difficult, especially at 98 DAS (20 days before the harvest). NDVI images were clustered according to k-means cluster analysis by QGIS Semi-classification plug-in to rapidly delineate subfield areas. The semi-automatic classification plug-in showed high potential and enabled quick image classification using clustering algorithms. Tempa and Aryal [53] found an overall accuracy of 85.5% of k-means unsupervised heterogeneous on land classification. Furthermore, cluster analysis for the subfield classification was used by many authors on different crops [[63], [64], [65], [66], [67]]. The thematic NDVI cluster layer starting from inflorescence development (53 DAS) to the ripening stage (98 ZS) identified three clusters grouped according to Cluster 1 (High), Cluster 2 (Medium) and Cluster 3 (Low) NDVI values. The Sentinel-2 derived –NDVI cluster maps through the k-mean statistics have clearly identified the three areas with higher, medium and low yield production. The NDVI mean value differences from the sentinel-2 time series map from Inflorescence development to the development of fruit were 22% lower from cluster 2 than in cluster 1, and 47% lower from cluster 3 related to cluster 1.

### 4.2 Ground yield data variability

The sunflower field of 29 ha showed a significant within-field yield spatial variability; the crop yields of georeferenced sampling data ranged from 1.3 to 3.77 t ha^−1^, with a yield reduction, in some areas, of 50% compared to the average yield values collected at harvest and maximum values approximately 40% higher than the average production values. The within-field yield variation at harvest agrees with what other authors have found in other cultivation environments on sunflowers [15,31]. High variability was recorded by the aboveground biomass, head diameter, stem diameter, and plant numbers. Aboveground biomass data align with findings by other studies like Claverie et al. [28]. As the plant population increased, head and stem diameter decreased significantly, following the findings by Sedghi et al. [68]. Georeferenced crop yield sampling was analyzed using a clustering method for assigning field information to potential subfield areas [66]. Three subfield areas were identified, and the highest yield value was recorded by cluster 1. Compared to cluster 1, cluster 2 had lower yield and yield trait values, with a 16% lower achene yield, 25% less dry biomass, and 20% lower head diameter. Cluster 3 showed a decrease in yield and yield traits of about 30% related to cluster 1. The most important agronomic factor affecting crop yields in the subfield areas with lower yields was the number of plants per square meter, which was higher than 40%, and accordingly, the diameters of the heads, the dry biomass and the stem diameters were lower by about 40% compared to the most productive ones.

### 4.3 NDVI data and yield traits

NDVI data vs yield and crop traits showed the best coefficient of determination for the fitted linear models at the flowering stage (R^2^ = 0.71) (63 DAT). Similar data were found by Amankulova et al. [15] and Dicu et al. [61]; when the sunflower was in the flowering stage, they discovered the strongest correlation between Sentinel-2 spectral reflectance and crop yield information provided by the combine harvester. Fieuzal et al. [31], found that the yield map obtained during the flowering stage presented spatial patterns consistent with those estimated just before harvest (regression close to 0.96 between the two estimated maps).

In this study, an R^2^ = 0.63 was found between Yield and NDVI, starting from the inflorescence development stage (53 DAS) to the ripening stage (88 DAS). The R^2^ values are slightly lower than those found by Narin and Abdikan [69], Inflorescence emergence stage on NDVI indices derived by Sentinel-2 images, providing a coefficient of determination (R^2^) higher than 0.67. Herbei and Sala [29] found that NDVI is the index most closely related with vegetation growth stages of sunflower crops. From the start of the vegetation stage until flowering, it had an inclining (ascending) slope. Peña -Barragan et al. [70] for the linear model calculated between yield and NDVI found an R^2^ = 0.6 at the early reproductive stage. Agüera Vega et al. [71], found a good correlation between NDVI and grain yield, aerial biomass, and nitrogen content in the aerial biomass, with the NDVI indexes acquired from the early reproductive stage to the flowering stage and found as the NDVI measured were not influenced by the hour when the images were taken, images can be acquired at any time of the day. The regression between NDVI and Yield value was lower than found by Tunca et al. [72], where an R^2^ of 0.91 was determined between NDVI values and yield. The results obtained confirm that there is an excellent correlation between production values and NDVI in the sunflower.

The best period for identifying homogeneous areas starts from the initial phase of the Inflorescences up to the development of fruit. Analyzing the Sentinel-2 NDVI k-means production by clusters and ground data, it is possible to state that cluster 1 covers an area of 42.4% of the total surface and 50% of the total achene yield; cluster 2 covers 35% of both surface and yield; instead the surface of cluster 3 covers 22.2% of the total surface with 15% of achene yield.

Future analysis of the pedo-environmental and climatic characteristics (exposure, soil, etc.) of these areas will lead to better management of the cultivation practices of the subfield areas with the management strategies of organic farming for increasing yield levels. Although increasing organic yields may negatively affect diversity [73], precision farming practices for increasing organic crop production can avoid the risk of attenuating the positive effects on biodiversity [13].

## 5 Conclusion

The proposed study addresses the potential of using NDVI derived from Sentinel-2 time-series for the delineation of subfield areas in sunflower crops. An unsupervised classification algorithm (k-means) was used to delineate subfield areas on the field based on a satellite-derived vegetation index. Ground data for within-field yield variation and spatial variability were recorded in the sunflower field. This study confirmed the high ability of the NDVI to detect the crop status of the sunflower starting from the inflorescence development stage. The cluster analysis of NDVI data from Sentinel-2 collected at nine crop growth stages elaborated by the Semi-automatic classification plug-in and the k-means algorithm has early delineated three sunflower subfield yield areas.

Analyzing the NDVI k-means maps with clusters and regressions determined from the field data, the best period for the identification of subfield areas starts from the inflorescences development stage up to the development of the fruit stage. Sentinel-2 data and k-means algorithms can improve an efficient assessment of subfield areas in sunflower crops. Identifying subfield areas can lead to site-specific long-term agronomic actions for improving the sustainable intensification of agriculture in the organic farming system.

## Author contribution statement

Stefano Marino: Conceived and designed the experiments; Performed the experiments; Analyzed and interpreted the data; Contributed reagents, materials, analysis tools or data; Wrote the paper.

## Data availability statement

Data will be made available on request.

## Additional information

Supplementary content related to this article has been publish online at [URL].

## Declaration of competing interest

The authors declare the following financial interests/personal relationships which may be considered as potential competing interests.

Stefano Marino reports financial support was provided by 10.13039/501100005401Ministry of Agricultural, Food and Forestry Policies (10.13039/501100005401MiPAAF). National strategic plan for the development of the biological system.
